# Insulin resistance in polycystic ovary syndrome across various tissues: an updated review of pathogenesis, evaluation, and treatment

**DOI:** 10.1186/s13048-022-01091-0

**Published:** 2023-01-11

**Authors:** Han Zhao, Jiaqi Zhang, Xiangyi Cheng, Xiaozhao Nie, Bing He

**Affiliations:** Department of Endocrinology, Shengjing Hospital, China Medical University, Shenyang, Liaoning 110000 People’s Republic of China

**Keywords:** Polycystic ovary syndrome, Insulin resistance, Hyperandrogenaemia, Insulin signal transduction pathway, Insulin sensitization therapy

## Abstract

Polycystic ovary syndrome (PCOS) is a common endocrine disorder characterized by chronic ovulation dysfunction and overabundance of androgens; it affects 6–20% of women of reproductive age. PCOS involves various pathophysiological factors, and affected women usually have significant insulin resistance (IR), which is a major cause of PCOS. IR and compensatory hyperinsulinaemia have differing pathogeneses in various tissues, and IR varies among different PCOS phenotypes. Genetic and epigenetic changes, hyperandrogenaemia, and obesity aggravate IR. Insulin sensitization drugs are a new treatment modality for PCOS. We searched PubMed, Google Scholar, Elsevier, and UpToDate databases in this review, and focused on the pathogenesis of IR in women with PCOS and the pathophysiology of IR in various tissues. In addition, the review provides a comprehensive overview of the current progress in the efficacy of insulin sensitization therapy in the management of PCOS, providing the latest evidence for the clinical treatment of women with PCOS and IR.

## Background

Polycystic ovary syndrome (PCOS) is currently recognized as the most common endocrine disorder in women of reproductive age, with a worldwide prevalence ranging from 6 to 21%, depending on the diagnostic criteria [1–5]. PCOS is a heterogeneous disease characterized by hyperandrogenism, dysfunctional ovulation, and polycystic ovary morphology, accompanied by metabolic abnormalities, such as insulin resistance (IR) and obesity. However, the underlying pathogenesis of PCOS remains unclear. Recent studies have suggested that genetics, epigenetic changes, environmental factors, oxidative stress, chronic low-grade inflammation, mitochondrial dysfunction, and metabolic disorders are involved in PCOS, thus damaging normal ovarian function [6–13]. IR and compensatory hyperinsulinaemia (HI) are considered major drivers of PCOS pathophysiology and are involved in the development of hyperandrogenaemia and reproductive dysfunction by various mechanisms [14].

IR and compensatory hyperinsulinaemia (HI) are present in 65–95% of women with PCOS, including the vast majority of overweight and obese women and more than half of women of normal weight. IR is independent of and exacerbated by obesity [14–18]. Currently, there are four commonly recognized phenotypes of PCOS: type A, polycystic ovary (PCO), chronic oligo-anovulation (OA) and hyperandrogenism (HA); type B, OA and HA; type C, PCO and HA; and type D, PCO and OA [19]. IR is present in all phenotypes, and insulin sensitivity varies according to the PCOS phenotype. IR is the most common classical phenotype (Types A and B) (80%), followed by ovulating PCOS (65%) and non-hyperandrogenaemic PCOS (38%) [20]. Women with PCOS and IR have a significantly increased risk of adverse pregnancy outcomes [21, 22] and chronic diseases, such as type 2 diabetes mellitus (T2DM), cardiovascular disease, and metabolic syndrome, which seriously affect the physical and mental health of women of childbearing age, increasing their social burden [23–25]. However, the root cause of IR in PCOS is largely unknown and the underlying mechanism remains to be elucidated. Therefore, recognizing the strong influence of IR on the occurrence and development of PCOS, accurate assessment of insulin sensitivity in the early stages of PCOS, and effective intervention on IR are essential to reduce the risk of long-term complications. Lifestyle change is the treatment of choice for all women with PCOS, and insulin sensitization is a promising choice for the chronic treatment of women with PCOS. This paper aims to summarize recent findings on the involvement of IR in the occurrence and development of PCOS and the mechanism of IR in various tissues. Furthermore, we aim to summarize and provide an update on the current research status of insulin sensitization therapy for women with PCOS to provide more effective and reasonable clinical treatment.

## Methods

An extensive literature search was performed up to July 2022 in PubMed, Google Scholar, Elsevier, and UpToDate databases. Keywords and subject terms included (“PCOS” AND “insulin”) OR (“PCOS” AND “insulin” AND “tissues”) OR (“PCOS” AND “insulin” AND “pathogenesis”) OR (“PCOS” AND “insulin” AND “diagnosis”) OR (“PCOS” AND “insulin” AND “evaluation”) OR (“PCOS” AND “insulin” AND “therapy”). Only English-language research papers were considered. In addition, publications focus on the new ones (since 2018) and exclude those without full manuscripts.

## Pathogenesis of insulin resistance in polycystic ovary syndrome

### Genetics and foetal origin

PCOS is an autosomal dominant genetic disease with various expression patterns that begins in early life, and metabolic changes precede reproductive abnormalities. A clustering analysis of 893 women with PCOS identified the metabolic subtype of PCOS, which was characterized by higher BMI and glucose and insulin levels with relatively low SHBG and LH levels [5]. IR is one of the prominent phenotypic characteristics of PCOS [26]. Twin and family cluster studies have suggested HI has a genetic component in PCOS, and a family history of T2DM is associated with significant insulin secretion defects [27, 28]. The daughters of women with PCOS develop HI and lower adiponectin levels before puberty [29], which persist throughout adolescence [30].

PCOS is associated with specific gene mutations, and most gene variants identified in genome-wide association studies are involved in regulating sheath steroid production, follicular maturation, or insulin signalling through the modified proteins they encode, such as insulin receptors, LH/HCG receptor activators, cell traffic proteins, and transcription factors [31, 32]. Genome-wide association studies on European, Chinese, and Indian populations have established that some insulin receptor (INSR) gene variants (rs2059807 and rs1799817) are significantly associated with IR in women with PCOS [33, 34]. Studies of Indian women suggest that C/T polymorphisms in the INSR tyrosine kinase domain may be susceptible variants in women with normal-weight PCOS, contributing to the development of IR and compensatory HI [35]. A meta-analysis showed that the Gly972Arg polymorphism in insulin receptor substrate 1 (IRS-1) mediates the pathogenesis of PCOS by increasing fasting glucose levels and is a risk factor for susceptibility to PCOS [36, 37]. However, the genetic assessment of insulin-related genes is affected by the diagnostic criteria and genotyping methods employed with patients, resulting in different results [38]. Exposure to adverse intrauterine environments can lead to varying degrees of IR and HI. Exposure to dihydrotestosterone and insulin in the second trimester of pregnancy produces a PCOS-like phenotype and increases the risk of miscarriage [39]. Intrauterine growth restriction can affect foetal insulin secretion, and insulin resistance trends in PCOS may be involved in developmental origin and preprogramming as a nutritional compensation mechanism [40, 41]. Adolescents and young women with a history of low birth weight are more likely than normal women to have PCOS-like manifestations of IR and high androgen levels [42, 43].

### Insulin signal transduction pathway

Insulin is a small peptide receptor-binding hormone released by pancreatic beta cells, which binds to cell surface receptors. INSR is a heterotetramer composed of α- and β-subunits linked by disulphide bonds. The extracellular α-subunit is responsible for binding to the ligands. The β-subunit is a glycoprotein spanning the cell membrane with tyrosine kinase activity [44]. Insulin binding to receptors induces specific tyrosine autophosphorylation, which phosphorylates intracellular substrates, including IRS1-4, SRC homologues, and collagen homologues (Shc), leading to a complex intracellular cascade that initiates insulin signal transduction [45]. Insulin has two main signalling pathways: metabolism and mitosis. Metabolism is primarily mediated through phosphatidylinositol 3-kinase (PI3-K) and serine/threonine kinase Akt/protein kinase B (PKB), also known as the PI3-K pathway. Through these pathways, insulin stimulates glucose uptake by promoting the translocation of glucose transporter 4 (GLUT4) from intracellular vesicles to the cell surface [46] and leads to the inactivation of serine phosphorylation of glycogen synthase kinase 3 (GSK3), increasing glycogen, fatty acid, and protein synthesis. It also activates mammalian target of rapamycin (mTOR) to regulate protein synthesis and degradation [46]. The mitotic pathway is the mitogen-activated protein kinase-extracellular signal-regulated kinase (MAPK-ERK) pathway, which is activated by insulin receptor-mediated phosphorylation of Shc or IRS. This progressively stimulates the translocation of cascade erk1/2 to the nucleus and phosphorylates transcription factors to stimulate cell growth and differentiation and regulate gene expression [47, 48]. Increased serine phosphorylation and decreased tyrosine phosphorylation of insulin receptors and IRS can terminate insulin action, resulting in post-binding defects in insulin signal transduction and leading to insulin dysfunction in women with PCOS [49, 50].

### Hyperinsulinaemia and tissue insulin resistance

**Fig. 1:**
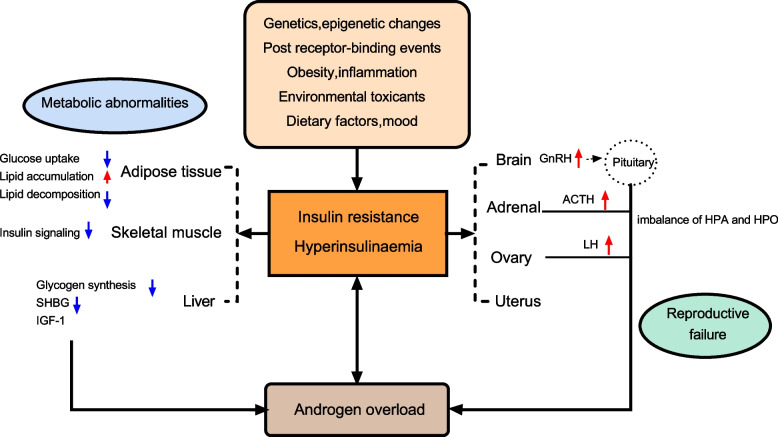
A summary of the most representative impact of IR and HI in women with PCOS. Abbreviations: SHBG: sex hormone-binding globulin; LH: luteinizing hormone; IGF1: insulin growth factor 1; GnRH: gonadotropin-releasing hormone; ACTH: adrenocorticotropic hormone; HPO: Hypothalamus-pituitary-ovary; HPA: Hypothalamus–pituitary–adrenal

IR in PCOS is caused by impaired insulin action in various target tissues, which is characterized by basal compensatory HI and a reduced insulin response to glucose overload. PCOS affects the majority of organ systems and tissues. Insulin plays different roles in different tissues in balancing the supply and demand of nutrients. HI caused by tissue IR is central to PCOS pathology [51]. IR in women with PCOS selectively and mutually affects metabolism or mitotic pathways in classical insulin target tissues (e.g., liver, skeletal muscle, and adipose tissue) and non-classical insulin target tissues (e.g., ovary, pituitary gland) [52]. In addition, androgen overload, lipid deposition, inflammatory cytokines, and other systemic factors are also involved in the IR process of peripheral tissues [53] (Fig. 1).

#### Adipose tissue

Adipose tissue plays a central role in systemic glucose metabolism and insulin sensitivity. Compared with body mass index (BMI)-matched control women, women with PCOS showed systemic fat accumulation and significantly increased subcutaneous fat cell volume, whereas visceral fat volume was only increased in PCOS phenotype A [54–56]. The increase in adipose tissue volume, especially visceral adipose tissue volume, is closely associated with whole-body IR [57]. The IR of adipose tissue results in decreased glucose uptake and lipid accumulation, and significantly inhibits lipid decomposition. Excessive fatty acids flow into skeletal muscle and liver, resulting in lipid storage and aggravating IR of skeletal muscle and the liver [58].

Adipose tissue IR in women with PCOS is influenced by circulating androgen levels and excessive energy intake. Androgens induce adipocyte IR by affecting the phosphorylation of insulin-stimulated protein kinase C (PKC), leading to a decrease in insulin-induced GLUT-4 content in PCOS abdominal subcutaneous adipocytes and a decrease in insulin-stimulated serine phosphorylation of GSK3, indicating the presence of insulin receptor binding or phosphorylation defects in adipocyte IR [59]. This impairs the effect of insulin on glucose metabolism but does not affect insulin-induced mitosis [59]. Androgens also regulate lipid metabolism and adipocyte differentiation, and induce the accumulation of abdominal adipose tissue. Animal models have shown that prenatal and postnatal exposure to androgens can lead to enlargement of adipocytes, accumulation of visceral fat, and decreased insulin sensitivity in women [60, 61]. In addition, testosterone is specific to catecholamine-stimulated lipolysis and can reduce the sensitivity of human subcutaneous fat cells but not visceral fat cells [62].

Type 1C3 aldosterone reductase (AKR1C3) is the only enzyme expressed in adipose tissue that can convert androstenedione into testosterone, which is widely expressed in the adipose tissue of patients with PCOS [63]. Studies have shown that AKR1C3 is the main driver of active androgen production in PCOS adipose tissue [63]. In addition, AKR1C3 expression is regulated by insulin [64], suggesting a significant correlation between testosterone and circulating insulin levels in adipose tissue. Increased androgen production in adipose tissue and subsequent lipid accumulation and fat mass can lead to systemic IR and lipotoxic organ damage in patients with PCOS [63]. HI further exacerbates hyperandrogenaemia, resulting in a vicious cycle that exacerbates poor metabolic performance [63]. In addition, free testosterone seen in PCOS is inversely proportional to the serum level of high-molecular-weight adiponectin, a collagen-like protein synthesized only by adipose cells, which has insulin sensitization and anti-inflammatory effects and decreases with adipose tissue volume [65]. Decreased adiponectin levels result in decreased PKC activity and insulin signalling [66]. Adiponectin also stimulates the secretion of hepatic sex hormone-binding globulin (SHBG), suggesting that the effect of androgen’on adipose IR may be influenced by hepatic SHBG through serum free testosterone levels [66]. Increased adipose tissue and its dysfunction may exacerbate physiological factors and cytokine levels, such as leptin, interleukin 6, and tumour necrosis factor alpha, thereby promoting low-level inflammation, interfering with insulin signalling, causing adipose tissue to release free fatty acids, increasing ectopic fat deposition, and aggravating IR on one’s own and other organizations [67–69].

#### Skeletal muscle

Skeletal muscle is responsible for most peripheral glucose uptake regulation, and almost two thirds of the glucose intake after meals is absorbed by skeletal muscle through insulin-dependent mechanisms, making it the most important insulin-resistant tissue [70]. Muscle insulin-mediated glucose processing, as measured by a normal glucose clamp, was significantly reduced in all women with PCOS compared to women without PCOS [71]. Skeletal muscle IR in women with PCOS is reflected by impaired insulin-stimulated glucose processing, which is a major risk factor for T2DM in women with PCOS [51]. However, human studies have not yet determined the molecular mechanism of PCOS-specific IR in skeletal muscle, and there have been many conflicting findings. The potential mechanisms currently considered include genetic and epigenetic programming, signalling pathway and mitochondrial dysfunction, intracellular and extracellular lipid accumulation, and organ system crosstalk [72].

Initial studies of muscle tissue and cultured myoducts and fibroblasts [73, 74] found elevated phosphorylation of serine residues on IRS1/2, resulting in the translocation of GLUT4 and reduced glucose uptake, suggesting defective signalling at the proximal insulin site. Skeletal muscle biopsies of women with PCOS revealed a significant decrease in insulin-mediated IRS1-related PI3-K activation, with an increase in IRS2 abundance as a compensatory change after targeted IRS1 destruction. Subsequent skeletal muscle studies showed decreased phosphorylation of Akt/PKB and the Akt substrate 160-KDA (AS160) [75]. These studies in obese women with PCOS suggest that possible PCOS-specific defect in insulin signaling of skeletal muscle is proximal and distal to IRS1/2. Hansen et al. studied molecular mechanisms in skeletal muscle underlying IR in normal-weight women with PCOS, finding that decreased insulin sensitivity may only play a role in skeletal muscle IR through AMPK and is associated with low circulating adiponectin levels [76]. In addition, a lack of insulin-stimulated pyruvate dehydrogenase activation in skeletal muscle may lead to reduced systemic metabolic flexibility and mediation of IR through metabolic signalling pathways [76]. Furthermore, constitutive activation of the mitotic signal MAPK-ERK1/2 has also been found in skeletal muscle biopsies of women with PCOS, which promotes serine phosphorylation of IRS1 and reduces metabolic signalling in PCOS myotube [77], suggesting that IR may impact both the metabolic and mitotic pathways in skeletal muscle of women with PCOS.

Animal studies have shown that hyperandrogenaemia promotes IR by increasing serine phosphorylation of Akt/PKB, mTOR ribosomal S6 kinase, and IRS1 in myotubes and promoting visceral fat accumulation [78]. In addition, hyperandrogenaemia may increase inflammation by activating nuclear factor kappa B (NF-κB), which in turn affects intracellular enzyme pathways associated with insulin receptors [51]. A recent meta-analysis suggested that obesity, but not HA or IR, appears to predict skeletal muscle mass in reproductive-aged women with PCOS [79]. Intramuscular lipid accumulation within muscle cells or between fibres may account for a small percentage of skeletal muscle IR [80]. PCOS is associated with abnormal skeletal muscle gene expression, and it is affected by specific changes in DNA methylation [72]. Furthermore, a link between TGF-β superfamily ligand signalling and extracellular matrix deposition in PCOS-specific IR results in inappropriate crossing between these cells and their host organs, suggesting that epigenetic as well as tissue crossing is involved in skeletal muscle metabolic abnormalities [72]. Mitochondrial oxidative phosphorylation genes are downregulated in the skeletal muscle of women with PCOS, suggesting that mitochondrial dysfunction is involved in PCOS-specific IR formation [72, 81].

#### Liver tissue

The liver is also the main site of glucose uptake and storage, accounting for one third of postprandial glucose processing, and the main site of insulin clearance [81]. Insulin in the liver promotes glycogen synthesis and de novo lipogenesis while also inhibiting gluconeogenesis. PCOS-related hepatic IR is usually only present in obese women, leading to a deficiency in insulin-stimulated liver glycogen synthesis and insulin-mediated inhibition of hepatic glucose production [82]. IR and compensatory HI can directly inhibit the synthesis of liver SHBG and insulin growth factor 1 (IGF1) binding protein. The former is a glycoprotein synthesized mainly in the liver, and its reduced synthesis leads to increased free testosterone levels [83]. The reduction of the latter increases the circulating concentration of IGF1, which not only triggers the ovarian membrane cells to produce more androgens, but also reduces specific microRNAs, thus accelerating the apoptosis of granulosa cells and inhibiting follicular development. These two effects together lead to hyperandrogenaemia and follicular development disorders in PCOS [13].

Insulin in the liver can directly regulate glucose and lipid metabolism and can also be indirectly regulated by fat and muscle insulin action. Direct effects activate de novo lipogenesis, convert excess carbohydrate substrates to triglycerides, and promote liver triglyceride delivery to adipose tissue [82]. Indirect effects are mainly caused by insulin-mediated inhibition of lipolysis in adipose tissue, which leads to an increase in circulating plasma non-fatty acids in the liver and promotes fat deposition in the liver. Approximately 59% of the lipids in hepatocytes are derived from non-fatty acids produced by adipolysis [82]. The liver-specific insulin receptor knockout (LIRKO) mouse model suggests that the insulin signalling is essential for the regulation of glucose homoeostasis in the liver and maintenance of normal liver function [84]; further, it is also a prerequisite for the indirect regulation of adipose insulin. LIRKO mice still showed obvious IR, severe glucose intolerance, and resistance to insulin’s ability to inhibit liver sugar production under the premise of normal insulin signal transduction in fat and muscle tissues [82]. In addition, hepatic lipid accumulation activates diacylglycerol/PKC and inhibits insulin receptors, affecting insulin signalling and subsequent gluconeogenesis, thereby exacerbating hepatic IR [53].

In vitro and in vivo studies of liver-specific androgen receptor (AR) gene knockout have found that AR signalling in liver cells mediates hepatic IR in hyperandrogenaemia-induced female mice through a cascade of changes in hepatic insulin signalling and phosphorylation, suggesting that androgens are involved in hepatic IR [85]. Long-term androgen excess can induce hepatic insulin resistance and steatosis in PCOS-like rats. Under hepatic IR, excessive androgens can promote the development of non-alcoholic fatty liver disease (NAFLD) through apoptosis and autophagy in the liver mitochondria [86]. NAFLD, a metabolic syndrome characterized by abnormal fat accumulation, is now considered the most common chronic liver disease in the United States, and its prevalence in PCOS has increased significantly in recent years [87]. Several human studies have shown a close association between NAFLD and liver IR, and IR is an important risk factor for NAFLD in PCOS [88–90].

#### The ovaries and uterus

Ovarian androgen overload is the core of PCOS. HI enhances intrathecal steroid production and leads to impaired follicular maturation. Insulin receptors are widely distributed in stromal and follicular ovarian cells, and there is considerable evidence for the direct ovarian effect of insulin on steroid production and the importance of insulin-signalling pathways in ovulation control [91, 92]. Under physiological conditions, insulin acts as a helper gonadotropin through its homologous receptor to increase LH-induced androgen synthesis in membrane cells, and LH induces luteinization in granulosa cells [93]. HI can lead to androgen-dependent anovulation via different mechanisms. Membrane cells are the main site of androgen production in the ovaries. Insulin acts on the membrane cells of the ovary to directly trigger androgen synthesis by increasing the activity of cytochrome P450c17α, a key enzyme that regulates androgen biosynthesis encoded by CYP17. Insulin can also cooperate with LH. The 17α-hydroxylase activity of P450c17 is activated by PI3-K signalling to induce androgen synthesis in membrane cells [94–97]. The membrane cells of women with PCOS are more sensitive to the hyperandrogenic effects of insulin than healthy women [51]. In anovulatory PCOS granulosa cells, the synergistic effect of high insulin and LH levels may induce premature expression of LH receptors in small follicular subsets, leading to premature differentiation of granulosa cells and follicular growth stagnation [98]. The effect of insulin on glucose metabolism was significantly reduced in granular lutein cells isolated from the ovaries of women with typical PCOS phenotypes, whereas the effect of insulin on steroid production was unchanged [98]. Reduced phosphorylation of MEk1/2 and MApK-ERk1/2 in PCOS was associated with increased P450c17 expression compared with that in normal membrane cells, contrary to the findings of increased phosphorylation of MEk1/2 and MApK-ERk1/2 in PCOS skeletal muscle [74]. These results suggest the existence of selective insulin resistance in PCOS ovarian tissue.

Energy metabolism is also critical for normal endometrial function, and endometrial studies of patients with PCOS have shown that IR and HI also negatively affect endometrial physiology. Endometrial tissues express molecules involved in insulin signalling pathways, and the expression of insulin receptors, IRS proteins, AS160, PKC, and GLUT4 in the endometrium of women with PCOS is impaired and associated with adverse reproductive outcomes [39]. Hyperinsulinaemia can impair decidualization of endometrial stromal cells in vitro through the transcriptional inhibition of FOXO-1 [99]. In addition, hyperandrogenaemia plays a role in the insulin signalling pathway of the endometrium, reducing the expression of INRS-1 and GLUT-4 in endometrial glandular epithelial cells [100]. The insulin sensitizer metformin promotes GLUT4 transcription by increasing AMPK, improves IR, and indirectly restores endometrial function in PCOS patients [101].

#### Central nervous system

Insulin affects the hypothalamic-pituitary system and can increase the frequency and amplitude of gonadotropin-releasing hormone (GnRH) release pulses through MAPK and increase GnRH gene expression, thereby increasing LH release, enhancing androgen biosynthesis in the ovary, and impairing ovarian function [102]. Insulin signalling in the central nervous system plays an important role in normal reproduction and body weight regulation. Female mice with neuron-specific destruction of IR genes show increased food intake, disrupted LH release, and impaired ovarian follicle maturation [103, 104]. Leptin, one of the earliest known adipokines, is essential in the hypothalamus for maintaining normal body weight and insulin sensitivity [105]. Specific knockout of insulin receptors and leptin receptors in hypothalamic proopiomelanocortin neurons induces a PCOS phenotype [106]. The pituitary gland is one of the most important components of the PCOS insulin. Insulin can directly stimulate LH secretion, leading to abnormal reproductive function in PCOS patients [107]. In addition, IR- and HI-mediated reduction of pituitary sensitivity to GnRH and disruption of pituitary insulin receptors can lead to anovulation [108]. Women with PCOS had a higher ACTH response to corticotropin-releasing hormone (CRH) stimulation than women who ovulate normally, which was strongly associated with HI severity [109]. IR and compensatory HI can increase adrenal androgen levels and aggravate PCOS hyperandrogenaemia by increasing adrenal sensitivity to ACTH [110].

Women with PCOS show hyperactivation of the AR in the preoptic area, hypothalamus, and other limbic structures [110]. Animal studies have found that male mice with neuronal AR deletions exhibit hypothalamic IR, suggesting that androgens may also promote IR by acting on the central nervous system [111]. In addition, increased leptin expression in the hypothalamus can aggravate obesity, and enhanced leptin secretion by adipocytes can further contribute to the induction of IR [48]. the two interact to cause metabolic disorders in PCOS.

### Factors affecting insulin resistance in polycystic ovary syndrome

Epigenetic changes (DNA methylation, histone status, and miRNA expression) are involved in the regulation of IR in women with PCOS. A study identified 79 differentially methylated genes in women who have PCOS with or without IR [112], and hypermethylation of the *LAMIN* gene promoter was associated with IR in PCOS [113]. MicroRNAs (miRNAs) are small non-coding RNA involved in the post-transcriptional regulation of genes. As regulators of gene expression, miRNAs are essential genes involved in the control of androgen synthesis, inflammation, adipogenesis, and signalling [114]. There are significant differences in miRNA expression levels between women with PCOS and healthy women [114]. Studies have shown that microRNAs secreted into the circulation by adipocyte exosomes and adipose tissue macrophages affect the PI3K/Akt-GLUT4 signalling pathway [115]. Mir-155-5p and related target genes of PCOS are concentrated in the insulin-sensitive pathway of the ovary, and Mir-222 is also positively correlated with serum insulin levels, suggesting their potential value as biomarkers of PCOS [115].

Insulin sensitivity can also be negatively affected by changes in diet, the environment, and mood. Recent studies suggest that an imbalance of intestinal flora and abnormal levels of metabolites produced by bacteria in individuals with may lead to insulin receptor signalling deficiency, leading to IR by causing immune system dysfunction, the development of chronic low-grade inflammation, and the enhancement of proinflammatory cytokine synthesis [116]. Vitamin D deficiency can also affect insulin signalling in tissues by affecting intracellular calcium regulation and exacerbating inflammatory responses [117, 118]. Melatonin is involved in regulating insulin secretion, and decreased melatonin secretion at night is associated with increased IR [119]. In addition, melatonin’s action is mediated by the melatonin receptor (MTNR), and activation of the MTNR1B signalling pathway in pancreatic beta cells reduces insulin secretion [120–122]. A meta-analysis also showed that MTNR1B RS1083096 and RS2119882 are involved in the pathogenesis of IR in Chinese women with PCOS [123]. Advanced glycation end products alter cellular translocation of insulin intracellular signalling and glucose transporters in PCOS through a variety of mechanisms, leading to tissue IR [124]. The endocrine disruptor bisphenol A also disrupts glucolipid metabolism and induces IR in PCOS by altering insulin secretion, adipocyte differentiation, and adipokine secretion [125]. In addition, chronic stress can trigger the release of cortisol from the hypothalamic–pituitary–adrenal axis, which can stimulate visceral fat accumulation, gluconeogenesis, and lipolysis, leading to IR [126, 127].

## Diagnosis and evaluation of insulin resistance in polycystic ovary syndrome

The glucose clamp technique is the ‘gold standard’ for evaluating metabolic insulin resistance in vivo. The amount of glucose injected in the steady state was equal to the amount of glucose absorbed by the peripheral tissue, which can be used to measure peripheral sensitivity to insulin [128]. Minimal model analysis using a frequently sampled intravenous glucose tolerance test (FSIGT) is an alternative to the simplified clamp procedure for assessing insulin secretion in insulin sensitivity experiments [129]. However, both the clamp test and FSIGT are complex, time-consuming, and expensive sampling procedures that are unsuitable for clinical practice. In recent years, clinical practice has developed many simple, cheap, and effective alternative quantitative indicators, such as BMI, waist circumference, waist-to-hip ratio, wrist circumference [130]and other anthropometric markers; fasting insulin, oral glucose tolerance test (OGTT), glucose/insulin ratio (G/I), homoeostasis model assessment of insulin resistance (HOMA-IR), quantitative insulin sensitivity test index (QUICKI) [131], lipid/lipoprotein ratio [132–134]and other biomarkers. These indices are reasonably correlated with each other and with the gold standard clamp technique. HOMA-IR is currently the best and most widely validated marker, but the cut-off point for the diagnosis of PCOS-IR is still not universally accepted [135]. Studies suggest that a more complex evaluation of the decrease in insulin sensitivity as a continuous variable is required in clinical practice [10]. In addition, owing to the strong association between inflammation and IR, inflammatory markers such as interleukin-6 (IL-6) [136] and ferritin [137] are becoming increasingly popular in the evaluation of IR, while cytokines such as leptin [138] and adiponectin [139] have also been proposed as new IR markers. However, conflicting data limit their use in clinical settings, and more studies are needed to clarify their suitability as IR markers in patients with PCOS [140].

## Treatment of insulin resistance in polycystic ovary syndrome

### Lifestyle change

Guidelines recommend that once women are diagnosed with PCOS and have decreased insulin sensitivity, they should make lifestyle changes and start insulin sensitivity treatment immediately, even if there are no significant changes in glucose tolerance [141]. The first step in managing IR is lifestyle change, which is the cornerstone of improving multiple endocrine and metabolic disorders in women with PCOS [142] and can be achieved through appropriate diet and exercise recommendations [143]. Studies on the relationship between caloric intake and expenditure in women with PCOS have been inconsistent, with preliminary data suggesting that the diets of women with PCOS tend to be high in carbohydrates and fat [144], with decreased satiety and increased sweet cravings [145]. Calorie-restricted diets may be the best option for reducing IR and improving body composition [146]. Studies have shown that the Mediterranean diet—which emphasizes a high intake of vegetables, fruits, seafood, legumes, and nuts; whole grains as staple foods; and the promotion of vegetable oils—combined with a low-carbohydrate regimen improves endocrine disorders and menstrual cycles in overweight patients with PCOS [147]. International evidence-based guidelines recommend that all women with PCOS, especially those who are overweight or obese, engage in at least 150 min of aerobic exercise per week, including more than 90 min of vigorous exercise [148]. There have been conflicting conclusions regarding the efficacy of the choice of optimal exercise mode in improving insulin sensitivity in women with PCOS. The heterogeneity of PCOS necessitates individualization of treatment options, and it appears that exercise combined with additional dietary/drug intervention is better for improving insulin sensitivity than either intervention alone [149–151].

Sleep deprivation is associated with an increased risk of IR, obesity, and T2DM in women with PCOS; therefore, sleep management should also be part of lifestyle change in women with PCOS [152]. Ensuring adequate, high-quality sleep can be an important initial change in women with PCOS. Since IR is strongly and independently associated with depression in PCOS, lifestyle interventions should be supported by mental health professionals who can provide appropriate psychological care for women with PCOS [25]. PCOS treatment is a long-term process, and diet and physical exercise require high self-discipline, are time-consuming, and are prone to relapse. Targeting IR is an effective strategy for treating PCOS. Clinical and experimental studies in recent years have explored several promising new therapies for improving IR in women with PCOS. Common pharmacological approaches for reducing IR in women with PCOS are outlined in the chart below (Table 1).

**Table 1:** Common pharmacological approaches for reducing IR in women with PCOS

Category	Generic Name	Ref	Mechanisms for improving insulin sensitivity	Common Side Effects	Contraindications
Biguanides	Metformin	[153–160]	↓gluconeogenesis ↓intestinal absorption ↓lipogenesis	gastrointestinal side effects (nausea, vomiting, diarrhea)	creatinine clearance < 40 mL/min
			↑glucose uptake ↑insulin receptor activity	headache	
TZDs	Pioglitazone	[161, 162]	↓lipidosis ↓fatty acid release ↓disruption of insulin activity by TNF α	upper respiratory tract infection、headache、myalgia	congestive heart failure
	Rosiglitazone	[163]	↑ target cell response to insulin ↑PPAR-γ transcription	peripheral edema	peripheral edema
GLP-1RAs	Liraglutide	[164, 165]	↑ glucose-dependent insulin secretion↑ lipolysis	nausea, vomiting	patients with a personal or family history of MTC
	Exenatide	[166, 167]	↓ body weight↓ lipogenesis	injection site reaction	multiple endocrine neoplasia syndrome type 2
	Semaglutide	[168]	↓oxidative stress ↓inflammatory response ↓Er stress	↑heart rate、hypoglycemia、headache	
DPP-4 inhibitor	Sitagliptin	[169]	↓ DPP-4 enzyme	upper respiratory tract infection	angioedema
			↑ incretin levels↑ insulin synthesis by pancreatic beta cells	nasopharyngitis、headache	
SGLT1/2is	Dapagliflozin	[170]	↓ reabsorption of glucose from renal tubules ↓renal threshold for glucose	increased urination、Female genital mycotic infections	severe renal impairment
	Empagliflozin	[171]	↓glucose toxicity and lipotoxicity ↓oxidative stress ↓inflammation		
	Linagliptin	[172]	↑ beta cell efficiency↑ caloric disposition ↑weight loss		
	Canagliflozin	[173]	↓ intestinal glucose reabsorption↑ secretion of incretin	mild diarrhea and nausea, urinary tract infection, Female genital mycotic infections	
Weight loss intervention	Orlistat	[174, 175]	↓ fat absorption ↓body weight ↓ gastric and pancreatic lipases	flatulence, steatorrhea, diarrhea, increased stool frequency	acute or chronic cholecystitis
			↓hydrolysis of dietary triglycerides to absorbable fatty acids	oily stool, fecal urgency	obstructive bowel disease
	Bariatric surgery	[176–179]	↑endogenous secretion of incretin and GLP-1	secondary hyperparathyroidism、gastric erosion	severe heart failure、coronary artery disease、esophageal varices
			↓ caloric intake by mechanically limiting food intake	Long term hypovitaminosis, increased bone fracture	stomach or esophageal ulcer、cirrhosis with portal hypertension
Supplements	Inositol	[180–185]	metabolic regulator ↑glucose uptake ↑glycogen synthesis and storage	not available	not available
	Alpha-lipoic acid	[186]	anti-inflammatory, antioxidant	not available	not available
	Omega-3	[187]	antioxidant、anti-inflammatory、anti-obesity ↑adiponectin	mild gastrointestinal distress, gas, nausea, diarrhea and headache	not available
	Coenzyme Q10	[188–190]	antioxidant, ↑glucose uptake	gastrointestinal distress	not available
	Vitamin E	[191, 192]	antioxidant	nausea, headache, blurred vision	not available
	Probiotics	[193–195]	↓inflammation, regulate intestinal flora and immune responses	not available	not available
	Carnitine	[196–199]	antioxidant, improves the β oxidation of fatty acids	not available	not available
TCM	Berberine	[200–202]	↑AMPK↑PI3K/Akt/GSK-3β↓MAPK ↓ lipogenesis ↑lipid consumption↑ antioxidase activity	gastrointestinal discomfort, constipation, mild abdominal pain, anorexia	not available
	Plant polysaccharides	[203, 204]	↑serum adiponectin, antioxidant	not available	not available
	Crocin	[205, 206]	anti-inflammatory; antioxidant ↑Glucose uptake	not available	not available
	Hehuan Yin decoction	[207]	↑PI3K/Akt/GSK-3β	not available	not available
	Acupuncture	[208–212]	↑GLUT4↑glucose uptake	slight bleeding at the acupuncture site	not available

Abbreviations: *PCOS* polycystic ovary syndrome, *IR* insulin resistance, *Ref* reference, *DPP-4* Dipeptidyl peptidase 4, *GLP-1* Glucose-like peptide 1, *GLUT4* Glucose transporter 4, *PI3K* phosphatidylinositol 3-kinase, *GSK* glycogen synthase kinase, *MAPK* mitogen-activated protein kinase, *AMPK* Adenosine Monophosphate Activated Protein Kinase, *MTC* Medullary thyroid carcinoma, *Er* endoplasmic reticulum, *TZDs* Thiazolidinediones, *SGLT1/2is* Sodium-glucose cotransporter type 1 and type 2 inhibitors, *GLP-1RAs* Glucagon-like peptide-1 analogues

### Insulin sensitization therapy

#### Metformin

Metformin, the most widely used insulin sensitizer for PCOS, reduces hepatic glucose production, inhibits gluconeogenesis and adipogenesis, improves peripheral tissue sensitivity to insulin, and prevents excessive insulin activity in the ovary [153]. Numerous studies have shown that metformin not only reduces weight and metabolic disorders but also corrects menstrual patterns, restores ovulation, and even improves chances of pregnancy [154]. Evidence-based guidelines recommend the use of metformin in obese, insulin-resistant women with PCOS to manage weight and endocrine and metabolic disorders, in conjunction with lifestyle adjustments [155, 156]. Metformin improves insulin sensitivity, alleviates metabolic disorders, and ameliorates polycystic symptoms in mice with PCOS by increasing the translocation of the glucose transporters GLUT1 and GLUT4 to cell membranes [157], activating the AMPK signalling pathway [158], and reconstructing the role of endogenous insulin-sensitizing molecules, such as adiponectin, in endometrial tissues under pathological conditions [159]. However, metformin use may be limited by gastrointestinal side effects [160].

#### Thiazolidinediones

Thiazolidinediones (TZDs) and peroxisome proliferator-activated receptor γ (PPAR-γ) agonists are true insulin sensitizers. PPAR-γ is a nuclear receptor that enhances insulin activity through a post-insulin receptor mechanism, primarily by improving insulin sensitivity in the adipose tissue and skeletal muscle [213]. TZDs can be used as alternative drug therapy for PCOS-related metabolic and reproductive abnormalities in women who cannot tolerate or respond poorly to metformin [161, 162, 213]. TZDs are effective as treatments for HI and IR in both lean and obese women with PCOS as well as in improving abnormal glucose tolerance, hyperandrogenaemia, and ovulation disorders in women with PCOS. Several studies and our mesh meta-analysis suggest that TZDs improve IR and dyslipidaemia in PCOS more than metformin does [163, 214, 215]. In addition, the combination of metformin and TZDs has a synergistic effect in the treatment of women with PCOS, conferring greater improvement in IR and menstrual frequency in PCOS than metformin alone [215]. Women with dyslipidaemia and PCOS may also consider rosiglitazone alone or in combination with low-dose metformin and lifestyle changes [119]. Compared to trioglitazone and rosiglitazone, pioglitazone shows a higher affinity for the specific receptor PPAR-γ, has more effective insulin sensitization and lower hepatotoxicity, but does not promote weight loss [109].

#### New antidiabetic drugs

Many new antidiabetic drugs have shown positive effects in the treatment of PCOS. Glucagon-like peptide-1 analogues (GLP-1RAs) mimic the incretin secreted by the distal small intestine, bind to insulin receptors on beta cells, stimulate insulin secretion, reduce glucagon secretion, inhibit hunger centres, and delay gastric emptying. They also exhibit anti-inflammatory properties [216, 217]. Recent studies have shown that GLP-1RAs therapy has excellent therapeutic effects in improving hyperandrogenaemia, increasing menstrual frequency, reducing manifestations of metabolic disorders such as obesity and IR, and reducing long-term cardiovascular risk in obese women with PCOS [164, 166]. The combination of GLP-1RAs and metformin appears to be superior to any single agent in reducing body weight, hyperandrogenaemia, IR, and ovulation disorders in women with PCOS and may even improve metabolic outcomes in women who previously had an inadequate response to metformin [162, 217]. However, most GLP-1RAs are administered subcutaneously. Simultaneously, correct administration of medication is crucial to reduce the occurrence of adverse reactions [164, 165, 167, 168, 218]. Sitagliptin, a DPP-4 inhibitor, enhances early insulin secretion by reducing incretin and GLP-1 degradation, reduces peak glucose after oral glucose intake in overweight women with PCOS, and improves visceral obesity [169].

Sodium-glucose cotransporter type 1 and type 2 inhibitors (SGLT1/2is) play important roles in glucose homoeostasis by reducing HI and improving IR by acting on glucose (heavy) absorption in the gut and kidney, respectively [219]. Although the mechanism of action of SGLT1/2is in PCOS has not been fully investigated, weight loss and HI, improved IR and glucose metabolism, and cardioprotective effects are beneficial in PCOS, suggesting that SGLT1/2is may be a novel treatment option [170–172]. In clinical trials, the SGLT2 inhibitor canagliflozin was not inferior to metformin in reducing HOMA-IR, and canagliflozin also significantly improved menstruation and body weight and reduced triglyceride levels, suggesting that it should be considered an effective treatment for patients with PCOS and IR [173]. Urinary symptoms are major adverse events associated with SGLT2 inhibitors, and more large-scale randomized controlled trials are expected to be published in the future to explore their therapeutic potential for PCOS [220].

#### Natural molecules and dietary supplements

Inositol is a compound with nine forms (sugar alcohols), of which inositol (MI) and d-chiral inositol (DCI) are the most abundant forms present in humans, playing important biological roles in mediating various effects of insulin. Several scientific studies have confirmed that it has excellent insulin sensitization efficiency in women with PCOS and promotes ovulation [180]. Given that inositol administration is safe and effective in ameliorating the reproduction and metabolism of patients with PCOS, it may be used not only as a treatment for infertile women but also as a preventive treatment during pregnancy [180–183]. Appropriate application of MI and a suitable proportion of DCI improves the health of organs and tissues, while long-term high-dose DCI monotherapy in women with PCOS has a negative impact [184]. Therefore, inositol treatment should be evaluated according to the specific situation and needs of patients, while its optimal ratio still needs to be further clarified and supported by large-scale clinical trials and pharmacokinetic studies to better adjust supplement doses [185].

Alpha-lipoic acid [186] and omega-3 fatty acids are two supplements that improve lipid and insulin sensitivity in women through their anti-inflammatory and antioxidant properties, although omega-3 fatty acids are high in calories [187]. Studies suggest that coenzyme Q10 has beneficial effects on glucose and lipid metabolism, insulin, HOMA-IR, and total testosterone levels in women with PCOS and can also improve ovarian function [188–190]. Vitamin E combined with coenzyme Q10 can improve IR and serum SHBG levels in PCOS [191, 192]. Supplementation with probiotics, prebiotics, and synbiotics in women with PCOS can improve IR, protect the intestinal barrier, and regulate the immune system, lipid profile, and other metabolic disorders [193, 194]. Recent studies have also found that a high-fibre diet consisting of whole grains, traditional Chinese medicinal foods, and prebiotics combined with the α-glucosidase inhibitor acarbose improved reproductive endocrine disorders, HI, and IR in women with PCOS compared with a high-fibre diet alone [195]. Animal studies have shown that aloe gel extract can act as a potential metabolic regulator of PCOS by controlling glucose homoeostasis, improving insulin secretion, and enhancing insulin-mediated glucose uptake to reduce glucose tolerance [221, 222]. Clinical trials have also shown that l-carnitine supplementation effectively improves PCOS-IR by improving fatty acid β-oxidation and carbohydrate metabolism [194, 195, 221]. A recent trial showed that metformin combined with pioglitazone and acetylcarnitine improved IR and ovulation in women with PCOS more than metformin plus pioglitazone [199].

#### Traditional Chinese medicine

Traditional Chinese medicine (TCM) is often an important complement to modern Western medicine. Compounds isolated from Chinese herbs are particularly beneficial in improving metabolic disorders. Berberine (BBR) is an alkaloid that can relieve IR and treat PCOS by acting on a variety of insulin signalling pathways, including PPAR, MAPK, and AMPK. In recent years, it has been regarded as a safe and effective insulin sensitizer, although clinical data are lacking. BBR in combination with metformin appears to improve insulin sensitivity more [200–202]. Plant polysaccharides have many different pharmacological effects, and *Dendrobium officinale* has been shown to be effective in alleviating IR in PCOS [203]. *Astragalus polysaccharides* may improve insulin sensitivity in PCOS model rats by upregulating serum adiponectin levels and it may play an important role in the treatment of IR [204]. In clinical trial studies, saffron had a significant protective effect on FBG, HOMA-IR, and inflammatory levels of women with PCOS [205, 206]. Pharmacological studies have also found that hehuan yin tang and yijing tang, as key components of various TCM prescriptions, can regulate androgen and insulin levels and improve PCOS-IR symptoms through various pharmacological pathways [207, 223].

Acupoint application was also effective in improving metabolism and IR in obese women with PCOS [205, 224]. Acupuncture is an important component of TCM. Systematic evaluation and some studies have shown that electroacupuncture may increase systemic glucose uptake and improve insulin sensitivity by activating the PCOS sympathetic nervous system and part of the parasympathetic nervous system [208–211], but it may not be as effective as metformin in improving insulin sensitivity in women with PCOS [212]. More large-sample clinical trials are needed to explore TCM as a potential treatment option for PCOS.

## Conclusion

In general, women with PCOS develop IR owing to abnormal insulin signalling and metabolic dysfunction in insulin-responsive tissues, with a high incidence of IR in PCOS and a significant negative impact on health. Here, we discuss the molecular mechanisms, diagnosis, and protocol of IR-based PCOS. The pathogenesis of IR in PCOS is not completely clear, and apparently includes genetic and epigenetic changes, deficiency of insulin signal transduction, hyperandrogenaemia, obesity, and inflammation. IR in different PCOS tissues can selectively affect metabolic or mitotic pathways in many tissues, including the ovaries. Therefore, effective prevention and treatment options should be evaluated to improve IR in PCOS patients. Lifestyle interventions and insulin sensitization therapy can be effective strategies for improving insulin sensitivity, while increasing ovulation and reducing androgen levels. Among all of the insulin sensitizers, metformin is the most widely used in PCOS. However, all mentioned drugs for PCOS are still off-label and further studies with larger sample sizes are needed to evaluate the efficacy of these new treatments and provide new insights into the molecular mechanisms of IR in PCOS. 

## Data Availability

All data generated or analysed during this study are included in this published article.

## Abbreviations

IR: Insulin resistance
PCOS: Polycystic ovary syndrome
HI: Hyperinsulinaemia
HA: Hyperandrogenism
OA: Oligo-anovulation
LH: Luteinizing hormone
INSR: Insulin receptor
PKB: Protein kinase B
BMI: Body mass index
FSIGT: Frequently sampled intravenous glucose tolerance
HOMA-IR: Homoeostasis model assessment of insulin resistance
TCM: Traditional Chinese medicine
LIRKO: Liver-specific insulin receptor knockout
NAFLD: Nonalcoholic fatty liver disease
SHBG: Sex hormone-binding globulin
GSK3: Glycogen synthase kinase 3
mTOR: Mammalian target of rapamycin
GLUT4: Glucose transporter 4
MAPK-ERK: Mitogen-activated protein kinase-extracellular signal-regulated kinase
PKC: Protein kinase C
MI: Inositol
DCI: D-chiral inositol
